# Integrating Network Pharmacology and Metabolomics to Decipher the Mechanisms Underlying the Therapeutic Effects of Wuzhi Dripping Pills Against MASLD

**DOI:** 10.3390/metabo16090601

**Published:** 2026-08-22

**Authors:** Huijun Wang, Miaoyuan Zhang, Kangkang Gao, Yaping Zhou, Aoxing Xiao, Jinyi Liu, Xiangjun Qiu

**Affiliations:** 1 School of Medicine, Pingdingshan University, Pingdingshan 467000, China; wanghuijun@pdsu.edu.cn (H.W.); 5873@pdsu.edu.cn (M.Z.); gaokangkang@pdsu.edu.cn (K.G.); 2 College of Basic Medicine and Forensic Medicine, Henan University of Science and Technology, Luoyang 471023, China; zhouyaping@stu.haust.edu.cn (Y.Z.); xiaoaoxing@stu.haust.edu.cn (A.X.); liujinyi@stu.haust.edu.cn (J.L.)

**Keywords:** Wuzhi Dripping Pills, MASLD, network pharmacology, metabolomics

## Abstract

**Background**: Metabolic Dysfunction-Associated Steatotic Liver Disease (MASLD) is a progressive condition predisposing to cirrhosis and hepatocellular carcinoma, with prevalence rising globally. Yet effective therapies remain limited. Wuzhi Dripping Pills (WZDP), derived from *Schisandra sphenanthera*, possess hepatoprotective and anti-inflammatory properties, though their efficacy against MASLD remains unexplored. **Methods**: Network pharmacology predicted active ingredients and targets, followed by KEGG enrichment analysis. A high-fat diet MASLD rat model was established to evaluate WZDP effects on weight, liver index, and serum markers. Serum metabolomic profiling via UPLC-MS/MS, combined with multivariate statistics and KEGG annotation, identified perturbed pathways. **Results**: Twelve bioactive compounds and 434 WZDP targets were retrieved, of which 74 intersected with 838 MASLD-associated genes. Enrichment implicated AGE-RAGE, insulin resistance, and HIF-1 signaling. In vivo, WZDP significantly improved anthropometric and biochemical parameters. Metabolomic analysis distinctly separated WZDP-treated from model groups, highlighting phospholipase D signaling as a key differential pathway. **Conclusions**: This integrative approach confirms that WZDP confers therapeutic benefits in MASLD through coordinated regulation of neuroactive ligand–receptor interaction, bile acid biosynthesis, phospholipase D signaling, and arginine–proline metabolism. Our findings furnish a molecular and metabolic rationale for further mechanistic studies and drug discovery efforts.

## 1. Introduction

Metabolic dysfunction-associated steatotic liver disease (MASLD), previously known as non-alcoholic fatty liver disease (NAFLD), is a heterogeneous group of chronic liver conditions characterized pathologically by hepatic steatosis, inflammatory infiltration, liver fibrosis and progressive hepatic injury [1]. As one of the most prevalent chronic liver diseases across the globe, MASLD continues to exhibit a rising global incidence trend [2,3]. Excessive lipid deposition in the liver can trigger lipotoxicity, further contributing to the initiation of MASLD. It may also advance to metabolic dysfunction-associated steatohepatitis (MASH), formerly termed nonalcoholic steatohepatitis (NASH), through the activation of multiple pathological mechanisms including endoplasmic reticulum stress, oxidative stress, organelle functional impairment and ferroptosis [4]. MASLD represents a continuous pathological progression spectrum, ranging from isolated simple hepatic steatosis to MASH, progressive liver fibrosis, cirrhosis and a series of subsequent severe complications [5]. Patients diagnosed with MASLD face a high risk of progressing to liver cirrhosis and hepatocellular carcinoma. Additionally, this disease elevates the susceptibility to multiple systemic disorders, such as type 2 diabetes mellitus, cardiovascular diseases, chronic kidney diseases and extrahepatic malignancies [3].

Accordingly, lifestyle modification remains the cornerstone of MASLD management [6]. Early interventions, including weight control and regular physical activity, have been shown to confer significant benefits. In parallel, the development of pharmacological agents that target insulin resistance, lipid metabolism, oxidative stress, inflammation, apoptosis, and fibrotic pathways has considerably expanded the therapeutic landscape for MASLD [7]. Over recent decades, herbal medicines have attracted increasing attention as promising candidates for the prevention and treatment of MASLD, largely due to their favorable safety and tolerability profiles, notable therapeutic effects, and low toxicity [8]. Of particular note, both single herbs and compound formulations have demonstrated substantial clinical promise against MASLD, exerting effects primarily through anti-inflammatory, antioxidant, lipid-lowering, insulin-sensitizing, gut microbiota-modulating, and anti-fibrotic mechanisms [9].

Wuzhi Dripping Pills (WZDP) represent an innovative modern preparation derived from the ripe fruits of *Schisandra sphenanthera*. In traditional medicine, this herb is documented to possess astringent properties, replenish *qi*, promote fluid production, and calm the mind [10]. Experimental evidence indicates that the dichloromethane fraction of *Schisandra sphenanthera* can alleviate diabetes-related abnormalities in rats, including hyperglycemia, dyslipidemia, elevated fasting insulin levels, and impaired glucose tolerance. Mechanistically, its anti-diabetic effects may involve modulation of the sweet taste receptor pathway, along with the PI3K/AKT/mTOR and AMPK/mTOR signaling cascades, thereby helping restore metabolic homeostasis [10]. Additionally, the petroleum ether fraction of this plant has been shown to improve type 2 diabetes mellitus through regulation of sweet taste receptors, correction of disturbed metabolite profiles, and alteration of gut microbial composition [11]. Lignans from *Schisandra* species are well-recognized hepatoprotective agents. For instance, lignans extracted from *Schisandra chinensis* have been reported to attenuate hepatic inflammation and steatosis in MCD diet-fed mice through modulation of the gut-liver axis [12]. Moreover, bioactive lignans isolated from *Schisandra sphenanthera*—the botanical source of WZDP—have demonstrated significant anti-inflammatory and anti-hepatic fibrosis activities [13], and the ethanol extract of *Schisandra sphenanthera* (Wuzhi tablet) has been shown to protect against cholestatic liver injury via activation of the PXR pathway and promotion of liver regeneration [14]. Collectively, these lines of evidence support the notion that WZDP may confer beneficial effects against MASLD.

Metabolomics enables the comprehensive profiling of endogenous small-molecule metabolites in biological systems, capturing the dynamic metabolic responses to pharmacological interventions. Unlike targeted approaches that focus on predefined pathways, untargeted metabolomics offers an unbiased snapshot of the global metabolic landscape, allowing the identification of unexpected pathway perturbations and novel biomarkers of drug efficacy. In the context of MASLD—a disease characterized by complex metabolic disturbances involving lipid, bile acid, and amino acid metabolism—metabolomics is particularly valuable for uncovering the multi-pathway regulatory effects of herbal formulations that are difficult to predict from genomic or proteomic data alone. Indeed, this approach has been widely adopted for elucidating the pharmacological mechanisms of medicinal plants and their compound formulations [15,16,17]. By integrating metabolomic findings with network pharmacology predictions, we aimed to not only identify the potential targets of WZDP but also validate its impact on downstream metabolic networks at the functional level.

In this context, the current study integrates network pharmacology, in vivo animal validation, and metabolomic profiling to systematically delineate the bioactive components and underlying regulatory logic of WZDP within the therapeutic framework for MASLD.

## 2. Materials and Methods

### 2.1. Chemicals and Reagents

Acetonitrile (LOT: A998-4), methanol (LOT: A452-4), and formic acid (LOT: A117-50) were purchased from Fisher Scientific (Waltham, MA, USA). Ammonium acetate (LOT: 73594) and ammonium formate (LOT: 70221) were purchased from Sigma-Aldrich (St. Louis, MO, USA). L-2-Chlorophenylalanine (mixed internal standard, LOT: C2001) was purchased from Shanghai Hengchuang Biotechnology Co., Ltd. (Shanghai, China). Succinic acid-d_4_ (internal standard, LOT: 293075-1G) was purchased from Sigma-Aldrich (St. Louis, MO, USA). L-Valine-d_8_ (internal standard, LOT: HY-I1124) was purchased from Shanghai Haoyuan Biotechnology Co., Ltd. (Shanghai, China). Cholic acid-d_4_ (internal standard, LOT: S22155-50mg) and D-luciferin (internal standard, LOT: S19260-100 mg) were purchased from Shanghai Yuanye Biotechnology Co., Ltd. (Shanghai, China)

WZDP (LOT: 230380) and diammonium glycyrrhizinate capsules (LOT: H10940191) were purchased from Nanchang Hongyi Pharmaceutical Co., Ltd. (Nanchang, China) and Chia Tai Tianqing Pharmaceutical Group Co., Ltd. (Lianyungang, China), respectively. Beijing Keao Feed Co., Ltd. (Beijing, China) supplied the ordinary feed used in this study. The specialized feed for MASLD induction was obtained from Jiangsu Collaborative Medical Biotechnology Co., Ltd. (Nanjing, China). According to the manufacturer’s specifications, the diet consisted of 23% casein, 0.39% L-cystine, 6.46% cellulose, 6.46% mineral mix, 0.13% vitamin mix, 0.26% choline bitartrate, 11% glucose, 14.15% maltodextrin, 2.53% peanut oil, 2.53% sesame oil, 28% margarine, and 5.09% taro-flavored milk tea powder by weight.

### 2.2. Network Pharmacology

Bioactive constituents of WZDP were screened from the TCMSP database using oral bioavailability (OB) ≥ 30% and drug-likeness (DL) ≥ 0.18, and their structures were verified through PubChem and DrugBank. The active ingredients potentially targeted were retrieved through TCMSP. After standardization using UniProt, these targets were intersected with MASLD-related targets from OMIM, DisGeNET, TTD, and GeneCards to obtain common targets. A network of drugs, ingredients, targets, and diseases was established in Cytoscape 3.9.1 (Cytoscape Consortium, San Diego, CA, USA), and topological parameters such as node degree centrality were calculated by Network Analyzer to determine the significance of every node within the network. The overlapping targets were uploaded to the STRING database (Homo sapiens) to construct and visualize a PPI network. Finally, GO (Gene Ontology) functional annotation and KEGG (Kyoto Encyclopedia of Genes and Genomes) enrichment analysis of the shared targets were performed with Metascape. The top 10 GO categories and the top 20 KEGG pathways were displayed to elucidate the involved biological and metabolic routes (https://www.bioinformatics.com.cn/).

### 2.3. Animal Experiment

Sixty adult male Sprague-Dawley rats (weighing 190 ± 20 g) were provided by Huaxing Laboratory Animal Farm, Huiji District (Zhengzhou, China). The animal production license number was SCXK (Henan) 2024-0007. Approval for this study was granted by the Animal Laboratory of Henan University of Science and Technology (approval No. 202407002). The rats were fed under the following conditions: 12 h light-dark cycle with light on from 8:00 to 20:00; The temperature was kept at 22 ± 2 °C with a humidity of 50 ± 10%. Food was withdrawn for 12 h prior to the experiment, but water was allowed, and all animal procedures were conducted in accordance with the Guidelines for Ethical Review of Laboratory Animal Welfare (GB/T35892-2018, Standardization Administration of the People‘s Republic of China: Beijing, China, 2018).

After a 1-week acclimatization period, the rats were randomly allocated to six experimental groups using a random number table to ensure unbiased distribution. The six groups (each group containing 10 rats) were: blank control group (designated as group A), model group (group B), low-dose WZDP treatment group (group C, receiving 172.5 mg/kg), medium-dose WZDP treatment group (group D, at 345 mg/kg), high-dose WZDP treatment group (group E, administering 690 mg/kg), and a positive control group (diammonium glycyrrhizinate, DG, group F, at 45 mg/kg). Group A was fed ordinary feed and had free access to water. The other groups were fed a special high-fat diet for 8 weeks (with the daily feed amount calculated as 10% of body weight per week).

After 8 weeks, blood specimens were collected from rats’ tail veins per group to verify whether the MASLD model was successfully established. Blood samples were centrifuged to separate serum, which was stored at −80 °C. Subsequently, except for the blank control group, all other groups were switched to the same ordinary diet as the blank control group and received intragastric administration for 4 weeks. Group A (blank control) and group B (model) received physiological saline (5 mL/kg). The low-, medium-, and high-dose WZDP groups were administered WZDP suspension at doses of 172.5, 345, and 690 mg/kg, respectively. The positive control group received diammonium glycyrrhizinate solution at a dose of 45 mg/kg.

After treatment, the rats were weighed, and blood was harvested from the tail vein for the second time. Centrifugation of the blood samples was performed at 2500–3000 rpm for 10 min to collect serum, which was then used for biochemical analysis using a fully automatic biochemical analyzer. Meanwhile, serum from the blank control group, model group, and medium-dose WZDP group was frozen and stored for subsequent metabolomic analysis. The rats were then anesthetized, and the whole liver was removed and weighed.

All data are expressed as mean ± standard deviation (SD). Statistical analyses were performed using SPSS Statistics (version 22.0, IBM Corp., Armonk, NY, USA) and GraphPad Prism (version 8.0, GraphPad Software, San Diego, CA, USA). For multiple group comparisons, one-way analysis of variance (ANOVA) followed by Dunnett’s post hoc test was employed to compare each treatment group with Group A and Group B, respectively. A *p*-value of <0.05 was considered statistically significant.

### 2.4. Metabolomics

#### 2.4.1. Extraction of Metabolites

For metabolomic analysis, serum samples from the blank control group, model group, and medium-dose WZDP group were used, with six biological replicates per group (*n* = 6 per group). After permitting the samples to come to room temperature, each sample (100 μL) was combined with 400 μL of a protein precipitation solvent, which consisted of a 2:1 *v*/*v* blend of methanol and acetonitrile that had been cooled to −20 °C, along with 4 μg/mL of a mixed internal standard. The mixture was vortexed for one minute, subjected to sonication in an ice-water bath for ten minutes, and then incubated at −40 °C for two hours. After centrifugation at 13,000 rpm for twenty minutes at 4 °C, a 150-μL aliquot of the supernatant was collected and preserved at −80 °C until needed for analysis. Before injection, the preserved supernatant was thawed, diluted with 100 μL of 50% methanol, and centrifuged at 20,000× *g* for fifteen minutes at 4 °C. The final supernatant was subsequently transferred to a sample vial for UPLC-MS/MS analysis.

#### 2.4.2. LC-MS/MS Analysis

The gradient elution program was adapted from previously established protocols and has been routinely applied in our recent metabolomics studies [15,16,17], with minor optimizations to ensure optimal chromatographic separation for rat serum samples. For chromatographic separation, a Waters ACQUITY UPLC I-Class Plus system equipped with an ACQUITY UPLC HSS T3 column (100 mm × 2.1 mm, 1.8 μm) was used (Milford, MA, USA). The temperature of the column was maintained at 45 °C, with an injection volume of 3 μL and a flow rate of 0.35 mL/min. Mobile phase A was water containing 0.1% formic acid, while mobile phase B was acetonitrile. The gradient elution program was set as follows: 0–2 min, 5% B; 2–4 min, B increased linearly from 5% to 30%; 4–8 min, from 30% to 50%; 8–10 min, from 50% to 80%; 10–14 min, from 80% to 100%; 14–15 min, held at 100%; 15–15.1 min, B rapidly decreased from 100% to 5%; and 15.1–16 min, maintained at 5%.

For mass spectrometric detection, electrospray ionization (ESI) was applied in both positive and negative ion modes. Following UPLC separation, samples were ionized using a heated electrospray ionization (HESI) source and analyzed by a Thermo Q Exactive HF high-resolution mass spectrometer (Waltham, MA, USA). The ionization conditions were as follows: spray voltage, 3.8 kV (positive) and 3.0 kV (negative); capillary temperature, 320 °C; auxiliary gas heater temperature, 320 °C; sheath gas flow rate, 35 arb; auxiliary gas flow rate, 8 arb; probe heater temperature, 350 °C; and S-lens RF level, 50. MS1 full scans were acquired over a mass range of *m*/*z* 70–1050 at a resolution of 60,000 (at *m*/*z* 200), with an AGC target of 3 × 10^6^ and a maximum injection time of 100 ms. The total acquisition time was 15 min. Data-dependent MS2 scans were triggered for the top 10 most intense precursor ions per full scan. The MS2 resolution was set to 17,500 at *m*/*z* 200, with an AGC target of 1 × 10^5^, a maximum injection time of 50 ms, an isolation window of 2 *m*/*z*, and normalized collision energies of 10, 20, and 40 eV using HCD activation.

#### 2.4.3. Data Preprocessing

Raw data were preprocessed using Progenesis QI v3.0 software (Nonlinear Dynamics, Newcastle upon Tyne, UK), including baseline filtering, peak picking, integration, retention time correction, peak alignment, and normalization. Metabolite identification was based on retention time, accurate mass, MS/MS spectra, and isotope distribution by searching against HMDB, LipidMAPS, METLIN, and an in-house library (LuMet-Animal 3.0). The parent ion mass tolerance was set to 5 ppm for HMDB and LipidMAPS, and 10 ppm for LuMet-Animal and METLIN, with corresponding fragment ion tolerances of 10 ppm or 20 ppm. Metabolite identification confidence was classified according to the Metabolomics Standards Initiative (MSI) guidelines. The majority of identified metabolites were annotated at MSI Level 2 (putatively annotated compounds) based on accurate mass and MS/MS spectral matching. A small number of metabolites were assigned to MSI Level 3 (putatively characterized compound classes) based on accurate mass matching without confident MS/MS interpretation. No metabolites were confirmed at MSI Level 1, as authentic reference standards were not used for absolute verification. During data cleaning, ion peaks with a relative standard deviation (RSD) > 0.3 in QC samples or a missing value ratio > 50% in any experimental group were removed. Remaining missing values were imputed with half of the minimum intensity across all samples, followed by log2 transformation. Prior to PCA and OPLS-DA analysis, the data were normalized using probabilistic quotient normalization (PQN) to account for differences in sample concentration. Additionally, Pareto scaling was applied to reduce the relative importance of large signals while preserving the contribution of smaller, yet biologically relevant, metabolites, thereby improving the interpretability of the multivariate models. Finally, metabolites were classified into confidence levels based on qualitative scores and MS/MS spectral similarity, and those with high confidence were selected for subsequent analysis.

After the raw data were preprocessed, multivariate statistical analysis was performed using MetaboAnalyst (MetaboAnalyst, Montreal, QC, Canada). Unsupervised principal component analysis (PCA) was first applied to characterize the overall sample clustering and separation trends between groups. Supervised partial least squares discriminant analysis (PLS-DA) was then used to build an initial classification model, and orthogonal partial least squares discriminant analysis (OPLS-DA) was used to remove classification-unrelated noise, thereby maximizing metabolic differences between groups. R^2^Y (model interpretation rate) and Q^2^ (model prediction rate) were used to evaluate model quality. Reliability was confirmed by 70% cross-validation and 200 permutation tests, which helped guard against overfitting.

Differential metabolites were screened based on a Variable Importance in Projection (VIP) threshold of >1 from the OPLS-DA model, combined with univariate statistical analysis (*p* < 0.05). The VIP > 1 criterion is a widely accepted standard in metabolomics for identifying significant differential metabolites, and recent methodological studies have systematically evaluated this threshold in the context of untargeted LC-MS metabolomics data [18]. To further assess the robustness of our findings, we also applied Benjamini–Hochberg false discovery rate (FDR) correction, and metabolites with a q-value < 0.05 were considered as FDR-confirmed differential metabolites. The high concordance between the two methods (>85% overlap) confirmed the reliability of the identified metabolites and pathways. Such significantly distinct metabolites were then processed through the KEGG database for pathway enrichment analysis.

## 3. Results

### 3.1. Results of Network Pharmacology

#### 3.1.1. WZDP and MASLD Common Target Identification

**Figure 1:**
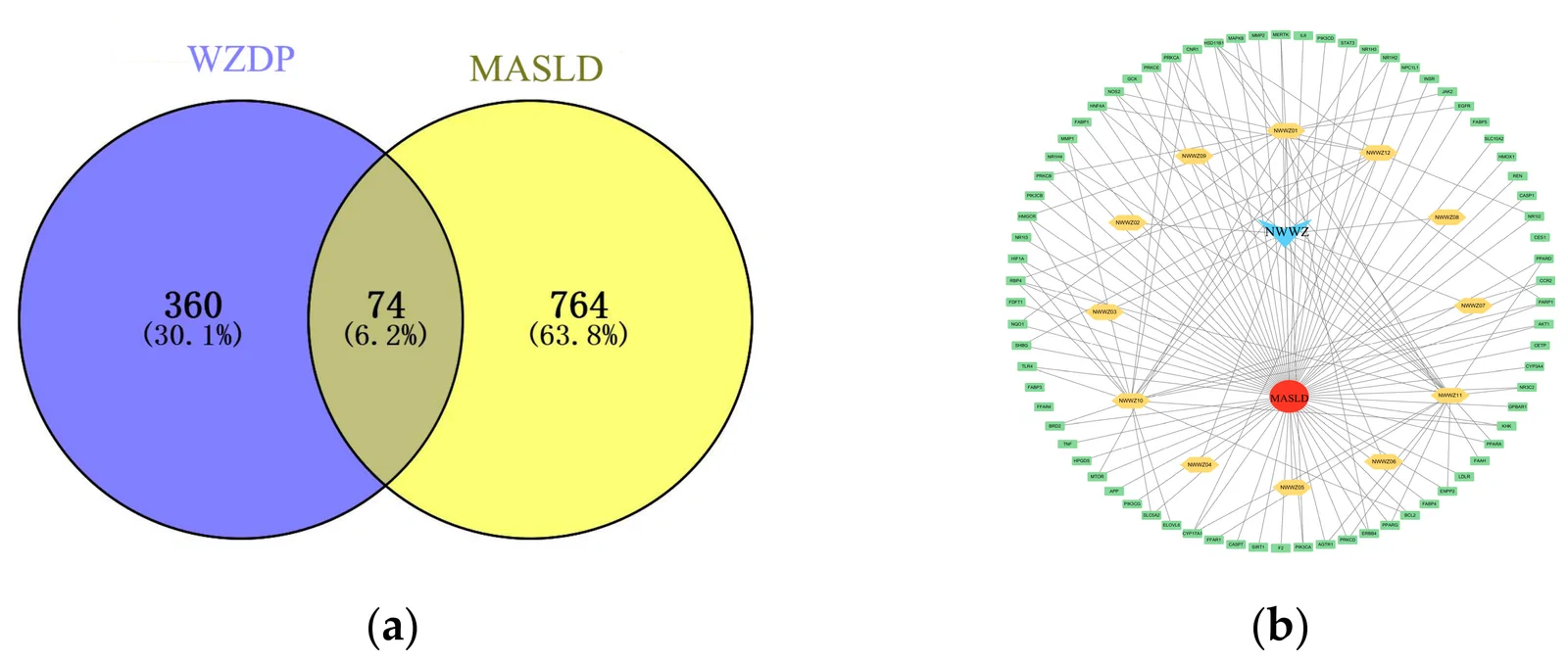

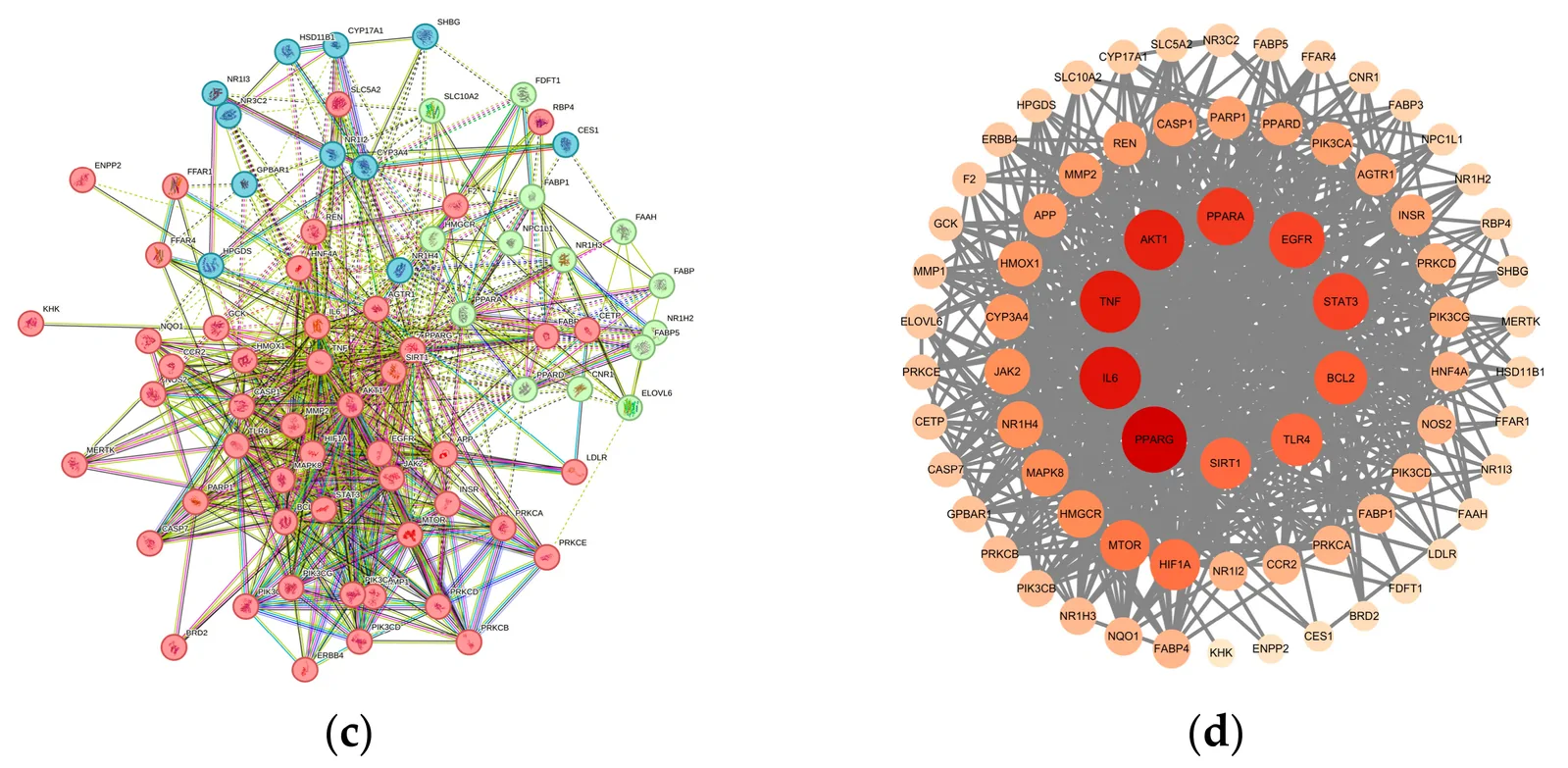
WZDP for the treatment of MASLD target Venny diagram (**a**), Active ingredient target disease network diagram (**b**), the protein–protein interaction network of the therapeutic targets of WZDP for MASLD (**c**), PPI image visualized by Cytoscape 3.9.1 (**d**). In (**b**), orange diamonds represent active ingredients, blue circles represent targets, and red hexagons represent the disease (MASLD). In (**c**,**d**), node size and color intensity are proportional to the degree centrality, with larger and darker nodes indicating higher connectivity.

Using the TCMSP database with criteria of OB ≥ 30% and DL ≥ 0.18, we identified twelve active ingredients that linked to 434 targets. Separately, 838 MASLD-related targets were obtained from the DisGeNET, OMIM, GeneCards, and TTD databases, resulting in a total of 74 overlapping targets (Figure 1a).

#### 3.1.2. Drug-Active Ingredient-Target-Disease Network

To explore the multi-component, multi-target mechanism of WZDP in the treatment of MASLD, a WZDP–active ingredient–target–MASLD interaction network was constructed (Figure 1b). Topological analysis showed that NWWZ01, NWWZ10, and NWWZ11 had the largest number of connected targets and were the core active ingredients; TNF, PPARA, and AKT1 had the highest degrees and were key hub targets. These findings clarify the material basis and core regulatory targets of WZDP against MASLD

#### 3.1.3. PPI Network

After obtaining the protein–protein interaction (PPI) network from STRING, the TSV file was downloaded and imported into Cytoscape 3.9.1, yielding 74 nodes and 648 edges. Following the selection of “Analyze Network”, the node size, transparency, and color were adjusted based on the degree value to generate the PPI network (Figure 1c,d).

#### 3.1.4. Enrichment Analysis

**Figure 2:**
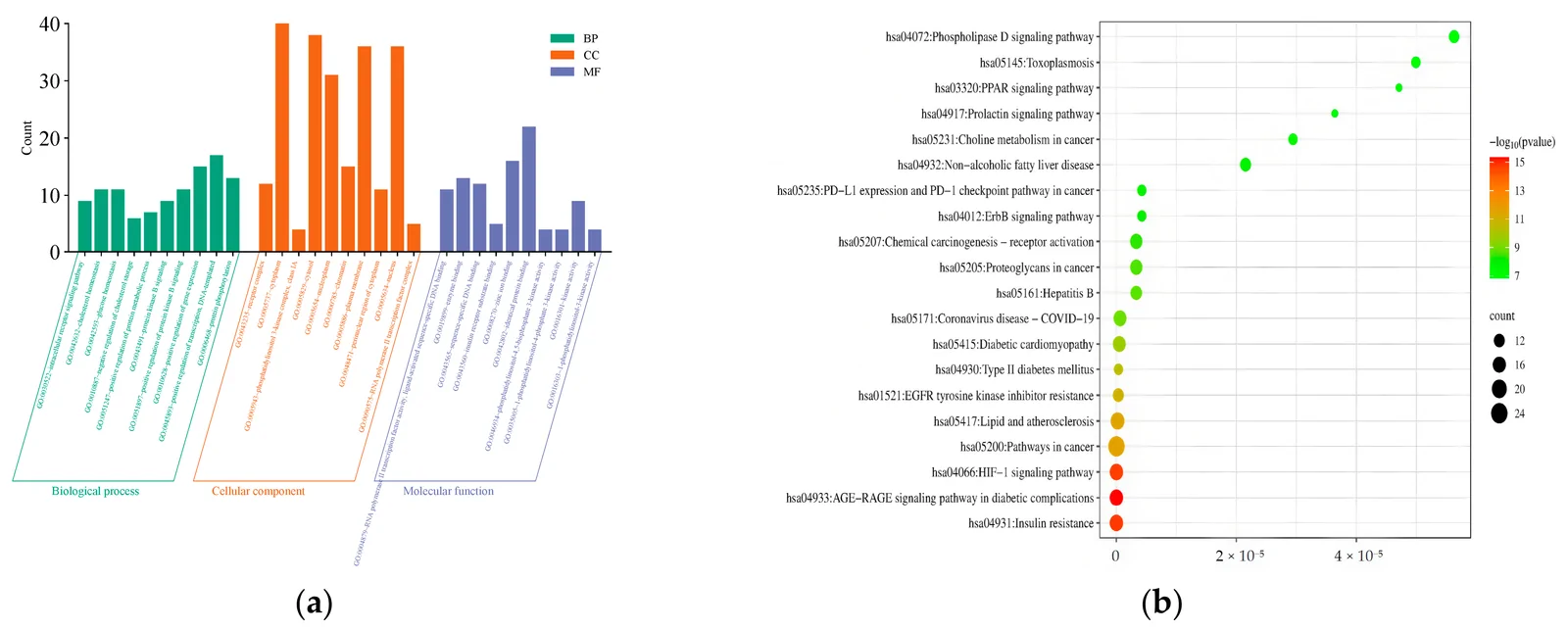
The GO bar chart of the therapeutic targets of WZDP for MASLD (**a**) and the KEGG bubble chart of WZDP for the treatment of MASLD efficacy targets (**b**).

GO functional annotation results (Figure 2a) showed that in terms of biological processes, the relevant targets of WZDP in treating MASLD were mainly involved in the intracellular receptor signaling pathway (GO:0030522), protein kinase B signaling (GO:0043491), glucose homeostasis (GO:0042593), and cholesterol homeostasis (GO:0042632). Regarding cellular components, most targets were found in the receptor complex (GO:0043235), phosphatidylinositol 3-kinase complex class IA (GO:0005943), cytosol (GO:0005829), and nucleoplasm (GO:0005654). Regarding molecular function, they were primarily associated with RNA polymerase II transcription factor activity, ligand-activated sequence-specific DNA binding (GO:0004879), insulin receptor substrate binding (GO:0043560), and sequence-specific DNA binding (GO:0003700).

KEGG pathway enrichment analysis (Figure 2b) revealed significant enrichment of the targets in the AGE-RAGE signaling pathway involved in diabetic complications (hsa04933), insulin resistance (hsa04931), the HIF-1 signaling pathway (hsa04066), and other pathways closely related to glycolipid metabolism and inflammation regulation. Among these, the phospholipase D signaling pathway (hsa04072) exhibited the highest degree of enrichment; in addition, the targets were also highly enriched in the PPAR signaling pathway (hsa03320), lipid and atherosclerosis (hsa05417), and non-alcoholic fatty liver disease (hsa04932), among other metabolism-related pathways. These findings suggest that WZDP may improve insulin resistance, regulate lipid metabolism, and inhibit inflammatory responses through the coordinated regulation of multiple pathways, thereby exerting therapeutic effects against MASLD.

### 3.2. Results of Animal Experiments

#### 3.2.1. Confirmation of MASLD Model Establishment

To confirm successful establishment of the MASLD model prior to drug intervention, baseline body weight and serum biochemical parameters were measured after the 8-week MASLD induction period. As shown in Table 1, rats in the high-fat diet groups exhibited significantly higher body weight compared with the control group (*p* < 0.05). Serum levels of ALT, AST, TC, and TG were also markedly elevated in the high-fat diet groups relative to the control group (*p* < 0.05 for all). These baseline data collectively confirmed that the MASLD model was successfully established, providing a valid foundation for the subsequent pharmacological evaluation of WZDP.

#### 3.2.2. Effect of WZDP on Body Weight and Liver Index in MASLD Rats

**Figure 3:**
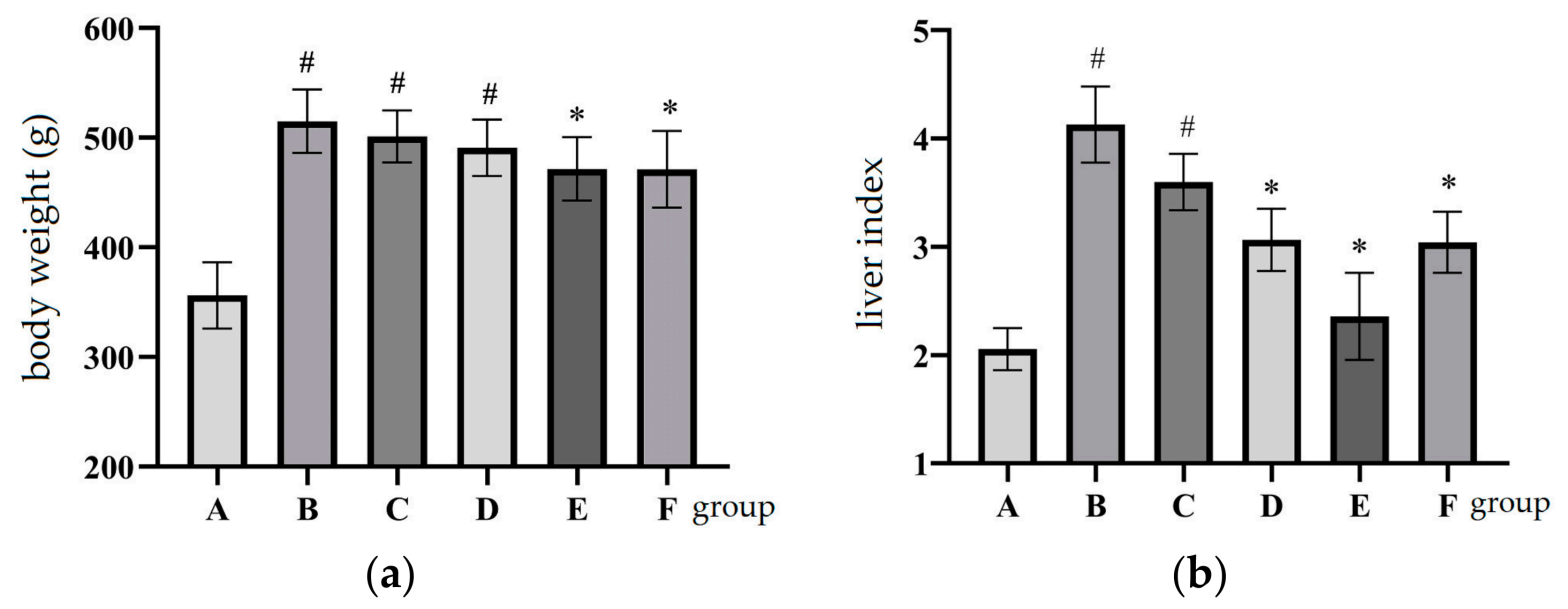
The body weight (**a**) and the liver index (**b**) in each group. # *p* < 0.05, vs. group A; * *p* < 0.05, vs. group B.

The MASLD model group showed a significantly higher body weight than the control group (Figure 3a,b), while it fell significantly in MASLD rats after high-dose WZDP or diammonium glycyrrhizinate treatment (*p* < 0.05). For the blank control group, the liver index (liver weight/rat weight × 100%) remained low, suggesting a healthy liver without obvious damage. In contrast, the model group showed a significantly increased liver index, indicating liver damage or pathological changes. After treatment, the liver index of each treatment group was markedly decreased relative to that of the model group, pointing to an improvement in liver condition. Specifically, treatment with medium-dose WZDP, high-dose WZDP, or diammonium glycyrrhizinate significantly decreased the liver index of MASLD rats (*p* < 0.05), with the strongest effect seen in the high-dose WZDP group.

#### 3.2.3. Biochemical Index Testing Results

**Figure 4:**
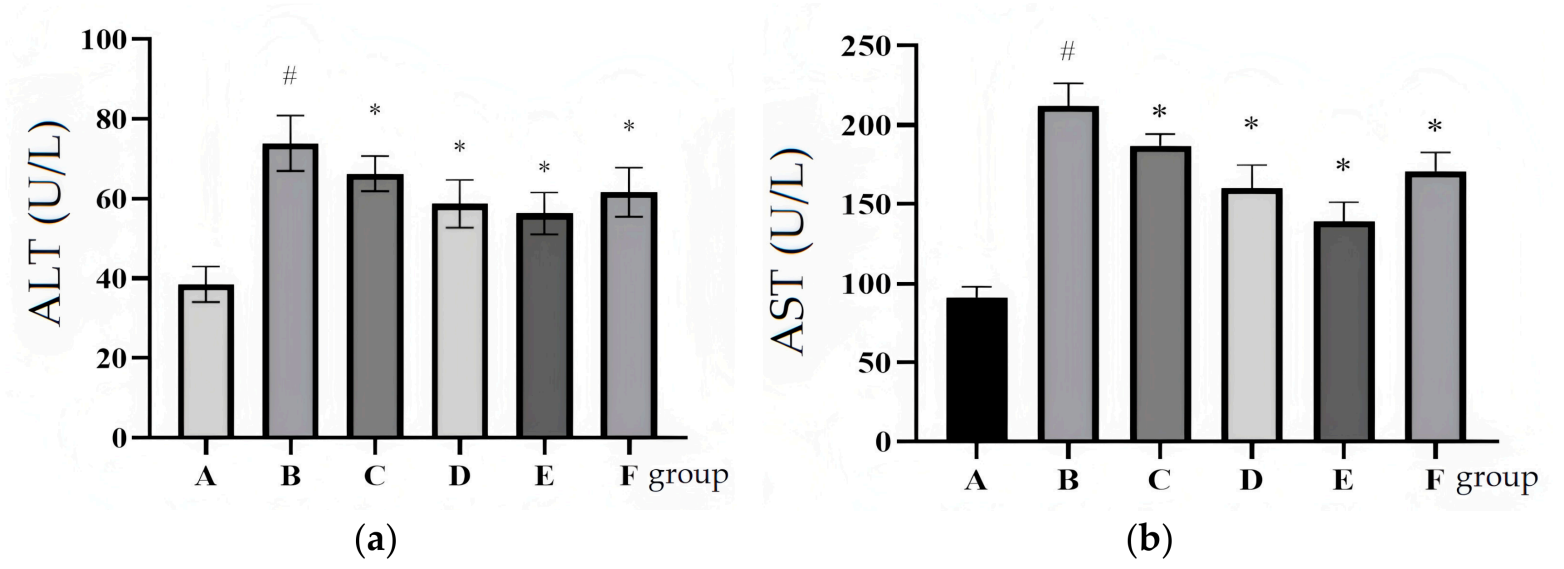

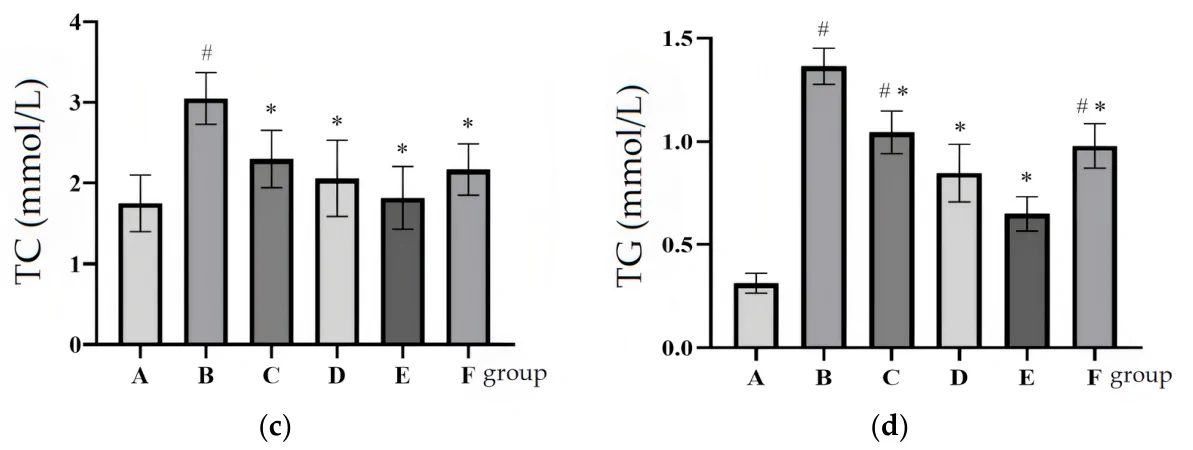
The ALT level (**a**), AST level (**b**), TC level (**c**), TG level (**d**) in each group. # *p* < 0.05 vs. group A; * *p* < 0.05 vs. group B.

As shown in Figure 4, multiple biochemical indicators in the serum of each group of rats changed significantly after different treatments. Compared with the blank control group, the alanine aminotransferase (ALT) and aspartate aminotransferase (AST) levels in the MASLD model group were significantly increased (Figure 4a,b), indicating marked hepatocellular damage. In contrast, ALT and AST levels were significantly reduced in all WZDP dose groups compared with the model group, with a greater reduction observed in the high-dose group. The positive control group (DG) also showed decreased ALT and AST levels, but the effect was less pronounced than that of the high-dose WZDP group. At the same time, the serum total cholesterol (TC) and triglyceride (TG) levels were significantly elevated in the MASLD model group (Figure 4c,d), suggesting lipid metabolism disorders. Blood lipid levels improved in all WZDP dose groups to varying extents, with the most significant effect observed in the high-dose WZDP group, where both TC and TG levels were markedly reduced. The positive control group also exhibited improved blood lipid levels, but the overall effect was inferior to that of the high-dose WZDP group. In summary, WZDP significantly improved various biochemical indicators in MASLD rats in a dose-dependent manner.

### 3.3. Metabolomics Analysis Results

#### 3.3.1. Evaluation of the Quality of the Experiments

**Figure 5:**
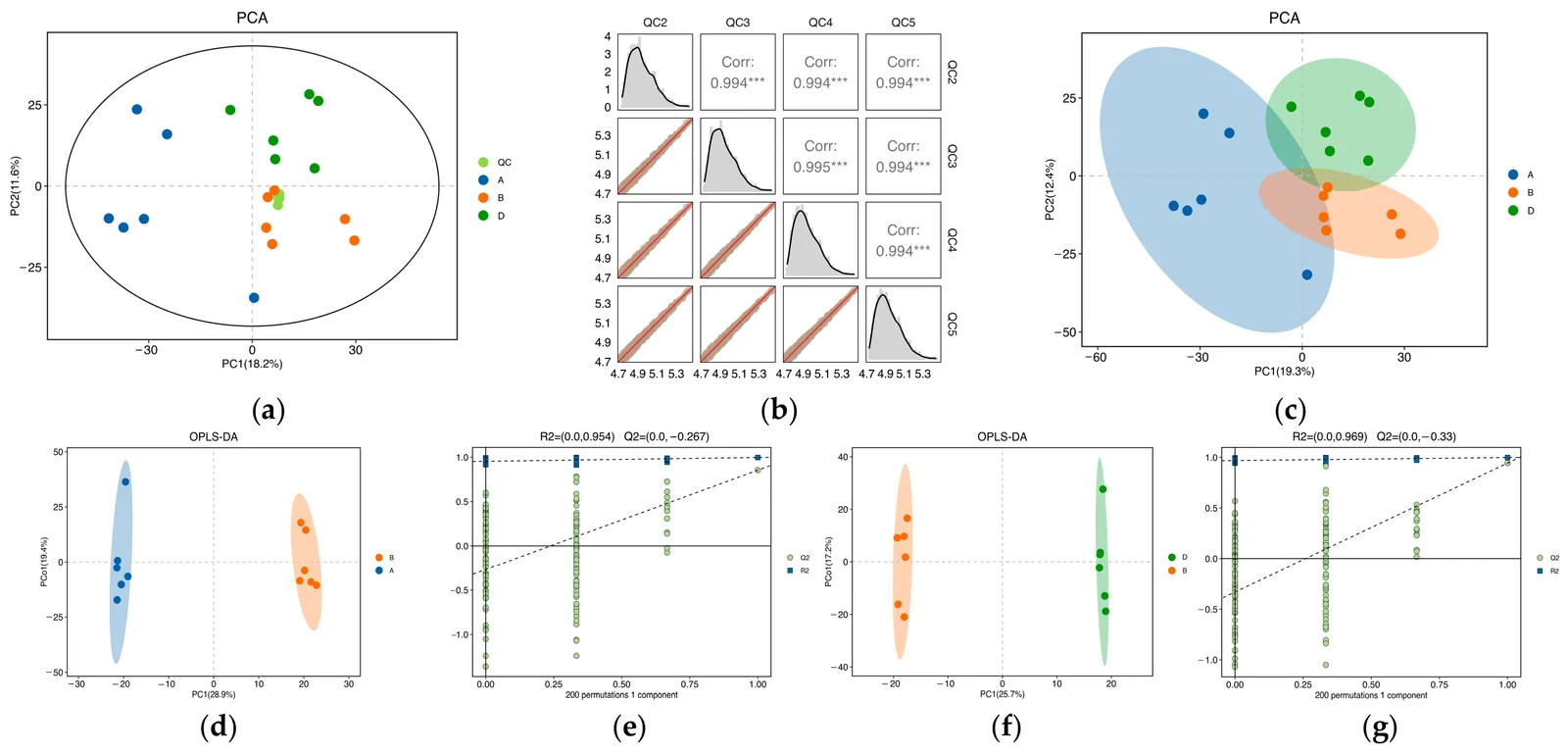
The PCA scores plot of all samples including quality control (QC) samples (**a**), the pearson correlation heatmap of QC samples (**b**), the PCA scores plot of samples from groups A, B, and D (**c**), the OPLS-DA score plot of comparison group A and B (**d**), the OPLS-DA permutation test plot of comparison group A and B (**e**), the OPLS-DA score plot of comparison group B and D (**f**), the OPLS-DA permutation test plot of comparison group B and D (**g**). In (**b**), the color gradient from blue to red represents the Pearson correlation coefficient (*r*), with blue indicating low correlation and red indicating high correlation. The numerical values shown in each cell represent the correlation coefficients (e.g., *r* = 0.994–0.995), and *** indicates *p* < 0.001, demonstrating highly significant correlations among QC samples.

QC samples served to monitor the entire experiment. In the PCA score plot (Figure 5a), QC samples formed a tight cluster, and the correlation heatmap (Figure 5b) showed good consistency among them, indicating high system stability and reliable data.

Multivariate statistical analysis clearly distinguished group A, group B, and group D. PCA (Figure 5c) separated the three groups, and the OPLS-DA score plot (Figure 5d,f) revealed distinct metabolic profiles between groups, with both R^2^Y and Q^2^ exceeding 0.5. The permutation test (Figure 5e,g) gave a Q^2^ regression line intercept less than zero. This suggests the model was not overfitted and was strongly reliable.

#### 3.3.2. Identification of Significantly Different Metabolites

The screening criteria were set as *p*-value < 0.05; metabolites with FC ≥ 1.2 were considered upregulated, and those with FC ≤ 1/1.2 were considered downregulated.

**Figure 6:**
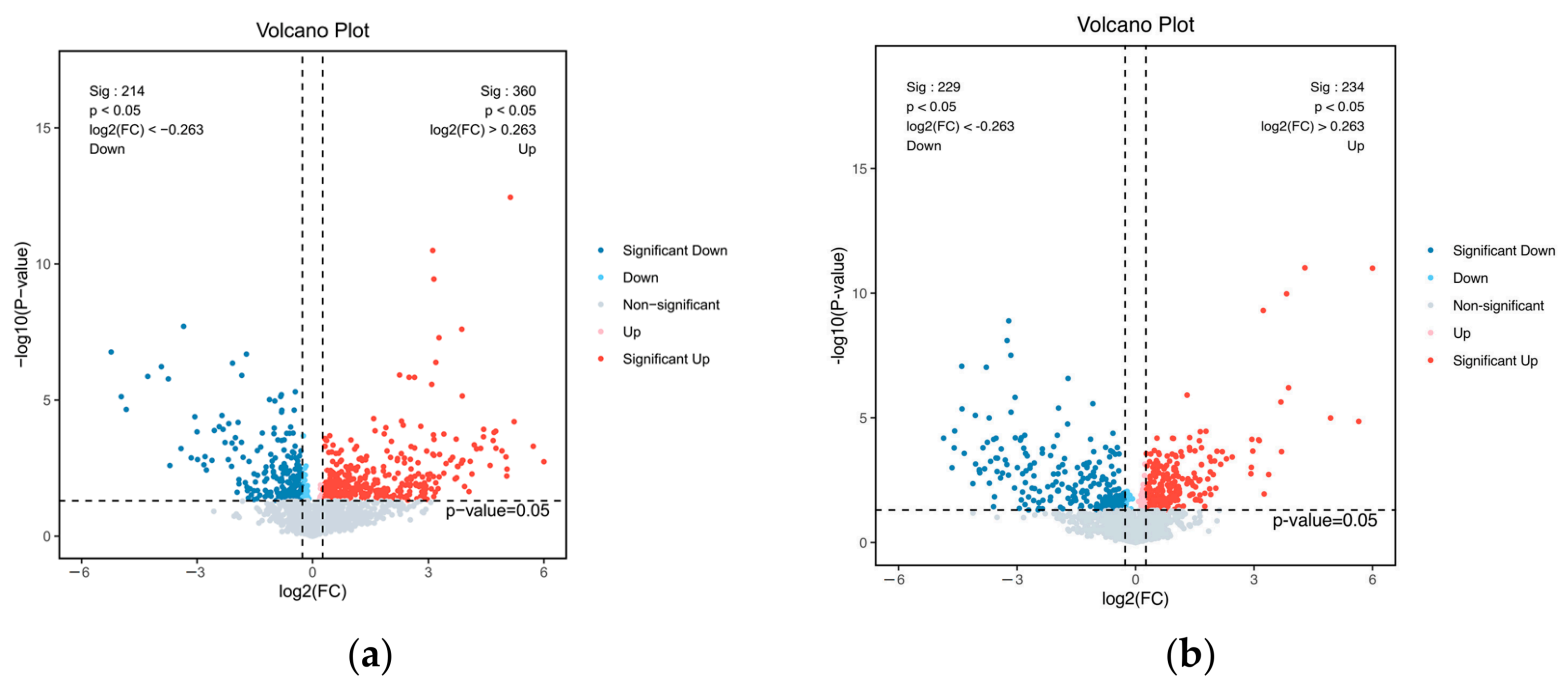
The volcano plot of differential metabolites between group A and group B (**a**), the volcano plot of differential metabolites between group B and group D (**b**). Red dots indicate significantly upregulated metabolites, blue dots indicate significantly downregulated metabolites, and gray dots indicate metabolites with no significant difference. Each dot represents an individual metabolite.

In positive ion mode, 211 significantly different metabolites were identified between group A and group B, of which 107 were upregulated and 104 downregulated. In negative ion mode, 363 differential metabolites were detected, with 253 upregulated and 110 downregulated (Figure 6a).

For the comparison between group B and group D, 169 significantly different metabolites were found in positive ion mode (87 upregulated, 82 downregulated). In negative ion mode, 294 differential metabolites were identified, with 147 upregulated and 147 downregulated (Figure 6b).

#### 3.3.3. Differential Metabolite Significance Analysis

**Figure 7:**
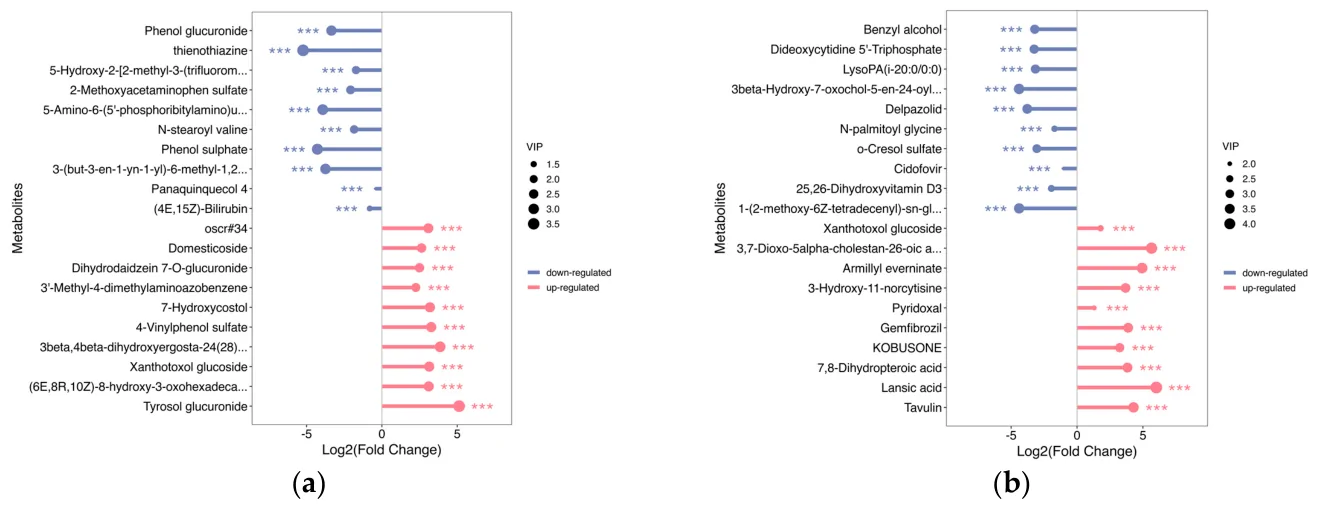
The comparison of the top 20 differential metabolites and VIP charts for group A and B (**a**), the comparison of the top 20 differential metabolites and VIP charts for group B and D (**b**). Metabolites marked with ‘***’ indicate VIP values greater than 2, suggesting particularly strong contributions to the group separation.

Figure 7 highlights the key differential metabolites in selected group comparisons. Here, the x-axis shows the log-transformed fold change (FC), and the y-axis lists the metabolite names. Blue dots on the left represent downregulated metabolites, and red dots on the right represent upregulated ones. Dot size follows the VIP value—bigger dot, higher VIP, more important. Metabolite names abbreviated with ellipses (“…”) in the Figure 7a are presented in full in the figure caption as follows (from top to bottom): 5-Hydroxy-2-[2-methyl-3-(trifluoromethyl)anilino]pyridine-3-carboxylic acid, 5-Amino-6-(5’-phosphoribitylamino)uracil, 3-(but-3-en-1-yn-1-yl)-6-methyl-1,2-dithiine; 3beta,4beta-dihydroxyergosta-24(28)-en-23-one, (6E,8R,10Z)-8-Hydroxy-3-oxohexadecadienoic acid. metabolite names abbreviated with ellipses (“…”) in the Figure 7b are presented in full in the figure caption as follows (from top to bottom): 3beta-Hydroxy-7-oxochol-5-en-24-oyl-glycine, 1-(2-methoxy-6Z-tetradecenyl)-sn-glycero-3-phosphoserine, 3,7-Dioxo-5alpha-cholestan-26-oic acid. In the comparison between group A and group B, Tyrosol glucuronide and thienothiazine are the most important upregulated and downregulated differential metabolites, respectively. Between group B and group D, lansic acid and 3β-Hydroxy-7-oxochol-5-en-24-oyl-glycine are the most important upregulated and downregulated differential metabolites, respectively.

#### 3.3.4. Differential Metabolite Abundance Analysis

**Figure 8:**
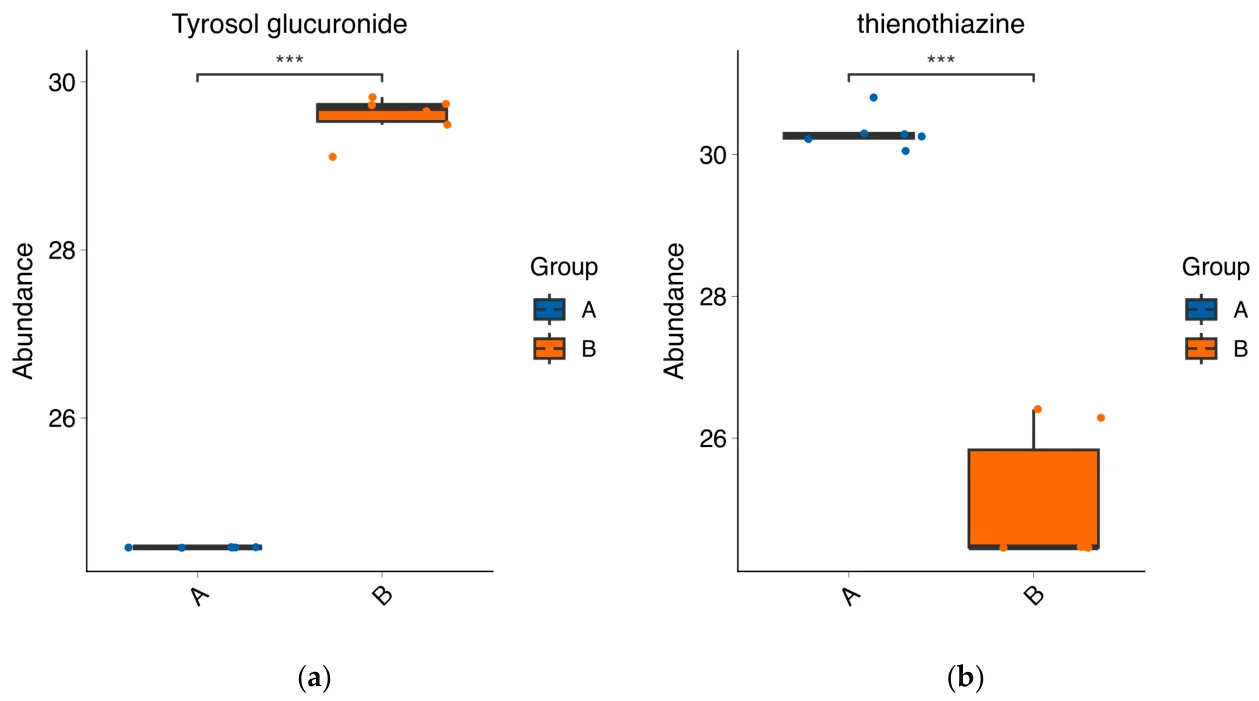

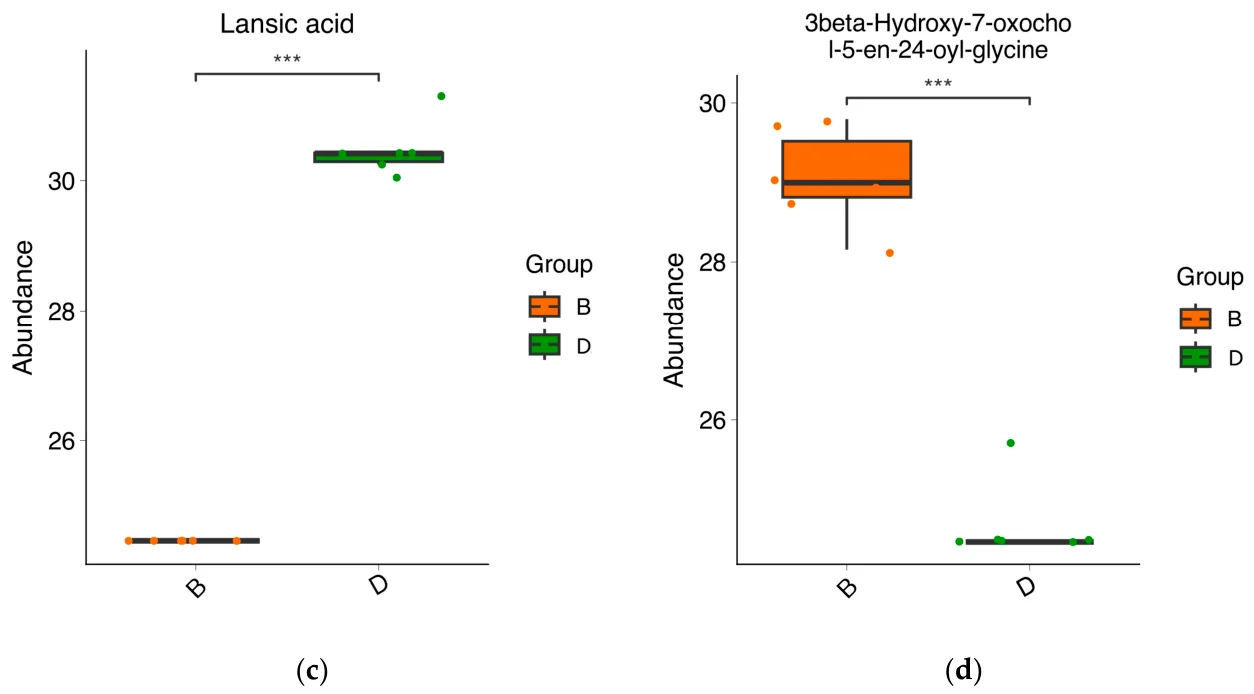
The shake point plots for tyrosol glucuronide (**a**), thienothiazine (**b**), lansic acid (**c**), and 3β-Hydroxy-7-oxochol-5-en-24-oyl-glycine (**d**). *** indicates *p* < 0.001 for the indicated pairwise comparison.

Figure 8 compares the average expression of four significantly different metabolites between the two groups, with groups on the x-axis and relative expression on the y-axis. After successful modeling, thienothiazine was down-regulated while tyrosol glucuronide was up-regulated in the model group. Following medium-dose WZDP treatment, 3β-Hydroxy-7-oxochol-5-en-24-oyl-glycine was down-regulated and lansic acid was up-regulated.

#### 3.3.5. Metabolomic Bioinformatics Analysis

To better understand metabolite differences among the three comparisons (AB, BD, and ABD), we mapped the identified metabolites to KEGG IDs and generated bubble plots for the top 20 enriched pathways, allowing a clearer visualization of metabolite functions and interactions.

**Figure 9:**
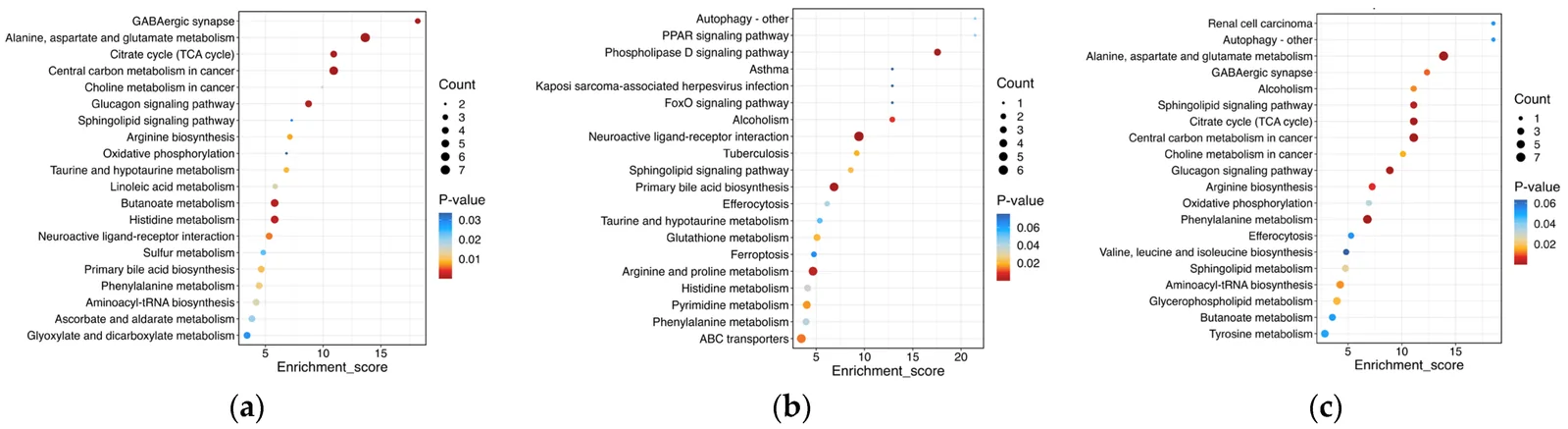
The VIP top 20 difference multiples and VIP charts of group A and B (**a**), group B and D (**b**), group A vs. B vs. D (**c**). The horizontal coordinate represents enrichment score and the vertical coordinate represents the pathway name; The size of the circle indicates count, i.e., the number of differential metabolites annotated to the pathway; the color of the circle corresponds to the corrected *p*-value, which is more significant from blue to red.

Comparing group A with group B (Figure 9a) reveals how MASLD directly affects metabolites, offering new clues for MASLD treatment. The enriched pathways include GABAergic synapse, alanine/aspartate/glutamate metabolism, and glucagon signaling—all pointing to MASLD-induced metabolic disturbances. Turning to group B versus group D (Figure 9b), we see how WZDP specifically alters metabolites in MASLD rats. Four pathways are highly enriched (*p* < 0.01): phospholipase D signaling, primary bile acid biosynthesis, arginine and proline metabolism, and alcoholism. Among these, the phospholipase D pathway shows the most pronounced difference, suggesting that WZDP may exert its therapeutic effects through these pathways. The comparison among group A, group B, and group D (Figure 9c) highlights pathways such as renal cell carcinoma and glutamate metabolism, reflecting the metabolic differences between healthy controls, MASLD model rats, and those receiving WZDP treatment.

Partial overlap between the KEGG pathways identified in the A vs. B comparison (disease-associated pathways) and those in the B vs. D comparison (WZDP-responsive pathways) suggests that WZDP targets specific therapeutic pathways for MASLD. By intersecting the enriched pathways from these two comparisons, we identified seven overlapping pathways that likely represent the core therapeutic targets of WZDP: neuroactive ligand–receptor interaction, sphingolipid signaling, primary bile acid biosynthesis, taurine and hypotaurine metabolism, histidine metabolism, and phenylalanine metabolism (Table 2). Among these, primary bile acid biosynthesis and taurine and hypotaurine metabolism were confirmed in the negative ion mode enrichment analysis, while the remaining pathways were identified in the positive ion mode, further supporting the robustness of these findings across complementary analytical platforms.

**Figure 10:**
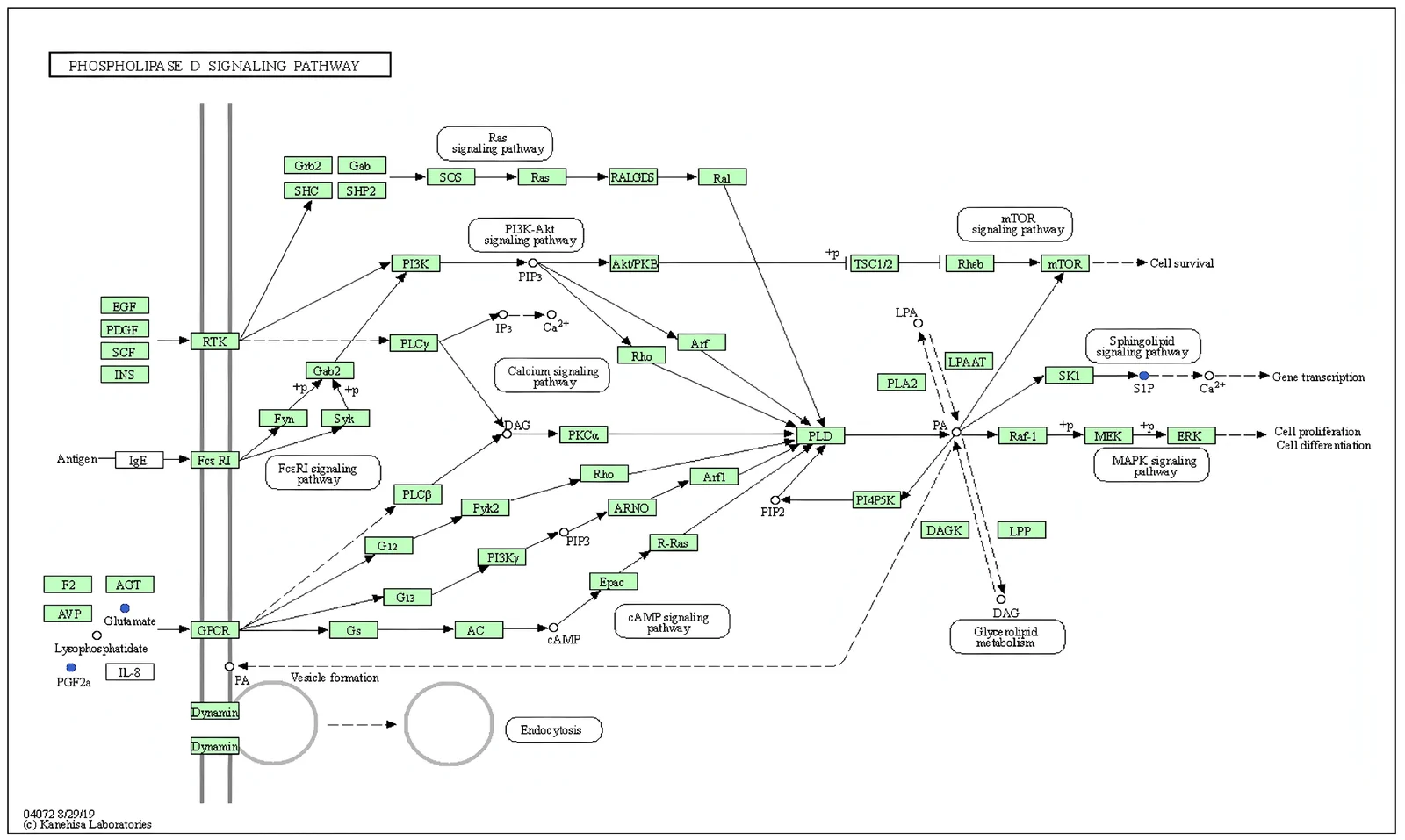
The KEGG mapping of the Phospholipase D signaling pathway. Key molecules and signaling cascades involved in the pathway are shown, including Ras signaling, PI3K-Akt signaling, calcium signaling, and Rho signaling. Green highlighted boxes represent enzymes or proteins identified as key targets in this study; blue circles represent differential metabolites identified in the B vs. D comparison. Solid arrows indicate direct activation or metabolic conversion; dashed arrows indicate indirect regulation or multi-step processes. Rectangles represent enzymes or proteins, and circles represent metabolites. The pathway map was generated using KEGG Mapper (https://www.kegg.jp/ accessd on 2 August 2026).

KEGG enrichment analysis of the phospholipase D signaling pathway (Figure 10), which was identified as a key pathway in the network pharmacology predictions, revealed changes in several key differential metabolites, including C00025 (L-glutamate), C00639 (prostaglandin F2α), and C06124 (sphingosine 1-phosphate). Notably, sphingosine 1-phosphate (C06124) is also a core component of the sphingolipid signaling pathway. This cross-pathway convergence suggests that WZDP may exert its therapeutic effects through coordinated regulation of interconnected metabolic networks involving phospholipid and sphingolipid metabolism. Although these metabolites were not identified as hits in the negative ion mode pathway enrichment analysis, their presence as differential metabolites and their topological importance within the PLD signaling pathway support their potential involvement in the pathogenesis or treatment of MASLD. However, their precise regulatory mechanisms require further investigation.

## 4. Discussion

This study integrates network pharmacology and metabolomics to explore the potential mechanism of WZDP in treating MASLD. Network pharmacology identified 74 core targets and enriched pathways, while metabolomics captured metabolic changes following drug intervention. The results of the two methods overlap but also differ. Specifically, network pharmacology indicates that WZDP may act through the AGE-RAGE, insulin resistance, and HIF-1 signaling pathways; metabolomics points to the phospholipase D signaling pathway, primary bile acid biosynthesis, and arginine/proline metabolism as key pathways. This discrepancy reflects the multi-target, multi-level complexity of drug action. Together, the two approaches complement each other and reveal the potential molecular mechanism of WZDP against MASLD.

Metabolomics results point to three key pathways through which WZDP may exert its therapeutic effects against MASLD. The phospholipase D (PLD) signaling pathway is central to cell signal transduction and participates in processes such as endocytosis, cytoskeletal remodeling, proliferation, survival, and lipid metabolism. PLD catalyzes the hydrolysis of phosphatidylcholine to produce phosphatidic acid (PA), a key second messenger that activates multiple downstream signaling molecules and engages in crosstalk with growth-promoting pathways such as Ras/Raf/MEK/ERK [19]. Recent studies have shown that PLD1 expression is significantly elevated in liver tissues of MASLD patients and high-fat diet-fed animals. Inhibiting PLD1 activity effectively reduces lipid accumulation in hepatocytes, possibly by suppressing PPARγ/CD36-mediated lipid uptake, improving insulin signaling, and influencing autophagic processes [20,21]. Thus, the PLD signaling pathway represents a promising therapeutic target for MASLD. The primary bile acid biosynthesis pathway converts cholesterol into bile acids, which are essential for lipid emulsification, absorption, and cholesterol homeostasis [22]. Bile acids also act as signaling molecules that activate the farnesoid X receptor (FXR) and participate in liver and intestinal metabolic regulation. MASLD is often accompanied by bile acid metabolism disorders; altered bile acid profiles and disrupted homeostasis promote hepatic lipid deposition and inflammation, and disease severity correlates with changes in the bile acid spectrum. Therefore, modulating this pathway is crucial for restoring metabolic balance [23]. Arginine and proline metabolism, as a key amino acid metabolic network, connects with the urea cycle and the tricarboxylic acid (TCA) cycle. Studies show that L-arginine supplementation can ameliorate metabolic syndrome features and regulate gut microbiota through its metabolite nitric oxide and polyamines, thereby alleviating MASLD progression [24]. Additionally, bioactive peptides containing the Arg-Pro-Arg motif have been reported to inhibit hepatic lipogenesis and improve lipid homeostasis, highlighting the regulatory potential of this pathway [25].

The integration of network pharmacology and metabolomics offers a more holistic view of WZDP’s therapeutic mechanisms. Network pharmacology pointed to key regulators of insulin signaling (AKT1) and lipid metabolism (PPARA) as primary targets of WZDP’s active components. In parallel, metabolomic profiling revealed that WZDP treatment notably affects phospholipase D signaling, bile acid biosynthesis, and arginine-proline metabolism. The convergence of these independent lines of evidence implies that WZDP’s lignan constituents may act through coordinated modulation of both upstream signaling cascades (e.g., insulin sensitization via AKT1) and downstream metabolic networks (e.g., phospholipid and bile acid homeostasis). This integrative framework helps translate target predictions into functional metabolic outcomes, thereby offering a mechanistic rationale for the multi-component, multi-pathway action of WZDP against MASLD. We acknowledge, however, that more rigorous quantitative integration—such as correlation analysis or pathway-level statistical modeling—was beyond the scope of this exploratory study and represents a worthwhile direction for future research.

In summary, the pathways predicted by network pharmacology (e.g., AGE-RAGE, insulin resistance) and those identified by metabolomics (e.g., phospholipase D, bile acid metabolism) collectively suggest that WZDP may treat MASLD by improving glycolipid metabolism, regulating bile acid homeostasis, and exerting anti-inflammatory effects. Future functional studies are needed to validate key targets and molecular mechanisms within these pathways, thereby providing a more comprehensive evidence chain for the use of traditional Chinese medicine in treating metabolic diseases.

## 5. Conclusions

Network pharmacology analysis demonstrated that WZDP exerts multi-target and multi-pathway therapeutic effects on MASLD, mainly through modulating the AGE-RAGE axis, insulin resistance signaling and the HIF-1 pathway. In vivo animal experiments further confirmed that WZDP treatment effectively corrected aberrant liver function and serum lipid profiles in MASLD-modeled rats. Mechanistically, the protective efficacy of WZDP against MASLD is closely associated with the modulation of key biological processes, covering phospholipase D signal transduction, bile acid synthesis and amino acid metabolism. Notably, the metabolism of arginine and proline serves as the core metabolic pathway underlying the pharmacological action of WZDP.

## Figures and Tables

**Table 1:** Baseline anthropometric and biochemical parameters after 8-week HFD induction for model validation.

Parameter	Control Group (*n* = 10)	Model Group (*n* = 10)	*p*
Body weight (g)	318.3 ± 29.8	527.7 ± 43.6	<0.01
ALT (U/L)	38.15 ± 3.84	79.59 ± 8.78	<0.01
AST (U/L)	90.11 ± 11.50	212.41± 21.74	<0.01
TC (mmol/L)	1.78 ± 0.48	3.12 ± 0.57	<0.01
TG (mmol/L)	0.34 ± 0.11	1.38 ± 0.16	<0.01

**Table 2:** Key pathway enrichment statistics for differential metabolites identified between the MASLD model group (B) and WZDP treatment group (D).

Pathway Name	Mode	Total	Hits	Raw P	FDR	Impact
Biosynthesis of unsaturated fatty acids	Neg	36	3	0.00035	0.02797	0
Purine metabolism	Pos	70	19	0.00015	0.01124	0.41986
Arginine biosynthesis	Pos	14	7	0.00038	0.01124	0.32447
Galactose metabolism	Pos	27	10	0.00042	0.01124	0.63032
Cysteine and methionine metabolism	Pos	33	11	0.00062	0.01239	0.53989
Fructose and mannose metabolism	Pos	20	8	0.00090	0.01446	0.59317
Tryptophan metabolism	Pos	41	12	0.00131	0.01744	0.20448
Alanine, aspartate and glutamate metabolism	Pos	28	9	0.00262	0.02894	0.60417
Pyrimidine metabolism	Pos	39	11	0.00289	0.02894	0.33768
Phenylalanine metabolism	Pos	8	4	0.00783	0.04475	0.76190
Pyrimidine metabolism	Neg	39	2	0.01137	0.41712	0.09246
Primary bile acid biosynthesis	Neg	46	2	0.01564	0.41712	0.01735
Taurine and hypotaurine metabolism	Neg	8	1	0.03471	0.69429	0.42857
alpha-Linolenic acid metabolism	Neg	13	1	0.05588	0.89412	0.33333
Arginine and proline metabolism	Pos	36	6	0.21841	0.56104	0.16744
Histidine metabolism	Pos	16	4	0.09870	0.32899	0.27049
Sphingolipid metabolism	Pos	32	3	0.72391	1.00000	0.00750

Note: Pos: positive ion mode; Neg: negative ion mode. Total: total number of metabolites annotated in the KEGG pathway; Hits: number of identified differential metabolites mapped to the pathway; Raw P: *p*-value from Fisher’s exact test; FDR: Benjamini–Hochberg adjusted *p*-value; Impact: pathway impact value derived from topology analysis based on metabolite centrality (MetaboAnalyst 5.0). Pathways with FDR < 0.05 were considered statistically significant; those with Impact > 0.10 were considered topologically important.

## Data Availability

The original contributions presented in this study are included in the article. Further inquiries can be directed to the corresponding author.
